# Prefabricated 3D-Printed Tissue-Engineered Bone for Mandibular Reconstruction: A Preclinical Translational Study in Primate

**DOI:** 10.1021/acsbiomaterials.1c00509

**Published:** 2021-11-22

**Authors:** Shuai-shuai Cao, Shu-yi Li, Yuan-ming Geng, Kausik Kapat, Shang-bin Liu, Fidel Hugo Perera, Qian Li, Hendrik Terheyden, Gang Wu, Yue-juan Che, Pedro Miranda, Miao Zhou

**Affiliations:** † Department of Oral and Maxillofacial Surgery, Guangzhou Key Laboratory of Basic and Applied Research of Oral Regenerative Medicine, Affiliated Stomatology Hospital of Guangzhou Medical University, Guangzhou 510182, China; ‡ Department of Oral and Maxillofacial Surgery/Pathology, Amsterdam UMC and Academic Center for Dentistry Amsterdam (ACTA), Amsterdam Movement Science, de Boelelaan, Vrije Universiteit Amsterdam 1117, Amsterdam, The Netherlands; § Department of Stomatology, Zhujiang Hospital, Southern Medical University, Guangzhou 510282, China; ∥ Department of Mechanical, Energy and Materials Engineering, University of Extremadura, Industrial Engineering School, Avda. de Elvas s/n, 06006 Badajoz, Spain; ⊥ Hangzhou Jiuyuan Gene Engineering Co., Ltd., Hangzhou 3100018, China; ○ Department of Oral and Maxillofacial Surgery, Red Cross Hospital, Kassel 34117, Germany; ∇ Department of Oral Implantology and Prosthetic Dentistry, Academic Center for Dentistry Amsterdam (ACTA), University of Amsterdam and Vrije Universiteit Amsterdam, Amsterdam 1117, The Netherlands; # Department of Anesthesia, Sun Yat-Sen Memorial Hospital, Sun Yat-Sen University, Guangzhou 510120, China

**Keywords:** mandibular reconstruction, 3D printing, TCP, PLGA/TCP, prefabrication, bone graft

## Abstract

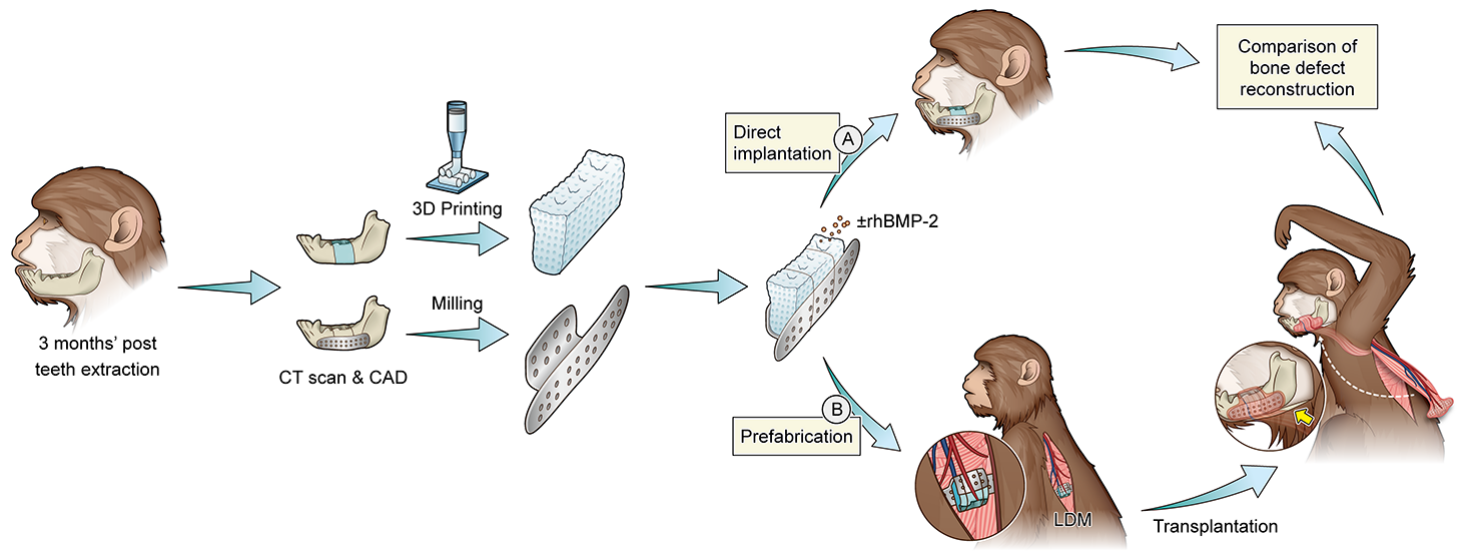

The advent of three dimensionally (3D) printed customized bone grafts using different biomaterials has enabled repairs of complex bone defects in various in vivo models. However, studies related to their clinical translations are truly limited. Herein, 3D printed poly(lactic-*co*-glycolic acid)/β-tricalcium phosphate (PLGA/TCP) and TCP scaffolds with or without recombinant bone morphogenetic protein −2 (rhBMP-2) coating were utilized to repair primate’s large-volume mandibular defects and compared efficacy of prefabricated tissue-engineered bone (PTEB) over direct implantation (without prefabrication). ^18^F-FDG PET/CT was explored for real-time monitoring of bone regeneration and vascularization. After 3-month’s prefabrication, the original 3D-architecture of the PLGA/TCP-BMP scaffold was found to be completely lost, while it was properly maintained in TCP-BMP scaffolds. Besides, there was a remarkable decrease in the PLGA/TCP-BMP scaffold density and increase in TCP-BMP scaffolds density during ectopic (within latissimus dorsi muscle) and orthotopic (within mandibular defect) implantation, indicating regular bone formation with TCP-BMP scaffolds. Notably, PTEB based on TCP-BMP scaffold was successfully fabricated with pronounced effects on bone regeneration and vascularization based on radiographic, ^18^F-FDG PET/CT, and histological evaluation, suggesting a promising approach toward clinical translation.

## 1 Introduction

Large mandibular defects originating from trauma and tumor ablative surgeries cause great suffering to patients due to compromised appearance and interruption of masticatory function and articulation. Vascularized autografts, such as fibular flaps, iliac bone graft, etc., are routinely used for mandibular reconstruction.^1^ However, donor site morbidity and difficulty in rehabilitating complex geometry and/or occlusal function of human mandible are still problems. With the development of 3D printing and tissue-engineering, customized tissue-engineered bone can be fabricated based on a 3D-printed bioscaffold fabricated from digital data from the human body.^3,4^

Conventional cell-based tissue engineered constructs seem to be compromised in repairing large bone defects due to limited vascularization and lack of a mature vasculature network, which are crucial for providing adequate nutrient and oxygen supply to the adhered/migrated cells beyond 200 μm from the nearest blood capillary.^2^ In these studies, a stable blood supply could be established in prefabricated tissue-engineered bones (PTEB) through endocultivation in a highly vascularized muscle pocket. However, bone overgrowth of PTEB was a serious problem, requiring further modification of PTEB to shape it before performing a reparative surgery.^6,7^ The advent of 3D printed metal, ceramic, polymer, as well as composite scaffolds mimicking complex geometries and mechanical properties of the native tissue greatly expedited maxillary reconstruction in small animals.^8−13^ Robocasting of customized β-tricalcium phosphate (β-TCP) scaffolds, often combined with certain chemicals or growth factors, could successfully repair large mandibular defects.^15−18^ However, these studies were mostly limited to the small animal models (such as rodents, rabbits, etc.), and their efficacy was not confirmed in large animals.

Among the nondestructive techniques, computed tomography (CT) can provide anatomical information on the defects. However, it cannot clearly distinguish the regenerated bone from the scaffold material owing to their similar gray scale value. For strong affinity toward bone apatite nanocrystals, ^99^Tc-methylene diphosphonate (^99m^Tc-MDP) was frequently used in single-photon emission computed tomography (SPECT) to trace bone metabolism, neo-bone formation, as well as vascularization.^19,20^^18^F-fluoride positron emission tomography (PET) was also successfully used to monitor BMP-2 release from calcium phosphate cement in rat calvarial defects and the sensitivity was more than SPECT due to a higher affinity of fluoride ions toward bone apatite.^21,22^

Glucose metabolism could be a potential target for real-time monitoring of BMP-2 mediated bone mineralization, since BMP-2 has an active role in glucose metabolism.^23−25^^18^F-Fluoro-deoxy-d-glucose (^18^F-FDG) is a well-known tracer in PET analysis, and this shares the same transporter system utilized for glucose uptake. Nevertheless, unlike glucose molecules, phosphorylated FDG (i.e., FDG-6-phosphate, a metabolite form of FDG) does not enter into the glucose metabolic pathway and gets accumulated inside cells.^26^ Supplementation of BMP-2 further increases the intracellular FDG-6-phosphate level. To the best of our knowledge, ^18^F-FDG PET was rarely explored for monitoring of BMP-2 activity for BMP-loaded tissue-engineered constructs.^27^

The present study aimed to explore the feasibility of customized PTEB constructs over directly implanted scaffolds toward faster recovery of mandibular bone defects. PTEB was constructed by ectopic implantation of BMP-2 incorporated/nonincorporated PLGA/TCP and TCP scaffolds in LDM and subsequently transferred with the pedicle to repair critical-sized mandibular defects of primates. Simultaneous bone formation and metabolic activities of the scaffolds were assessed by ^18^F-FDG PET/CT, and the recovery of bone defects was evaluated by radiographic, mechanical, and histological examinations. A schematic illustration of the study is provided in Figure S1. Inspired by the evidence-based treatments and pressing clinical needs, this study would open up new surgical methods and approaches toward sizable bone defect repair.

## 2 Materials and Methods

### 2.1 Design of Customized Implants and Titanium Mesh

For the design of customized implants, the tested animals were first induced with a general anesthesia and subjected to CT scan (Somatom 64-channel, Siemens, Munich, Germany) at 120 kV, 120 mA, and 0.625 mm slice thickness. The 3D model of the mandible was reconstructed using Mimics software v16.0 (Materialise, Leuven, Belgium), and the data was exported in digital imaging and communications in medicine (DICOM) format. A 20 mm-long defect of the mandible (in the edentulous region) was created by virtual osteotomy, and the retrieved data was imported into CATIA V5R21 (Dassault System, Vélizy-Villacoublay Cedex, France) to set porosity and pore size. The resultant data was optimized by Geomagic 13.0 (3D Systems, Rock Hill, South Carolina, United States) to generate STL files for 3D printing. The data file was further uploaded in a CAD program for 3D medical surface rendering (3DMSR, JIMAFEI Science and Technology Development Co. Ltd., China), and a virtual surgery was performed using virtual template along with a titanium mesh for internal fixation. The heights of the titanium mesh at buccal and lingual sides were maintained as 12 and 10 mm, respectively, while 1.2 mm uniform pore size was allocated to the screws.

### 2.2 Fabrication and Characterizations of PLGA/TCP and TCP Scaffolds

The polymeric template as a surgical cutting guide for mandibular surgery and the titanium mesh for scaffold fixation (as shown in Figure S1) were fabricated according to STL file using 3D polymer printer (Project 3510 HD Plus, 3D system, U.S.A.) and milling machine (GSVM6540, Gold Sun Mold & CNC machinery, China), respectively. The mandibular scaffolds of TCP and PLGA/TCP were fabricated by different 3D bioprinting techniques.

TCP scaffolds were fabricated by an established protocol, using a robotic deposition device (3-D Inks, Stillwater, OK, U.S.A.) fitted with Robocad 3.0 software (3D Inks, U.S.A.).^28^ β-TCP powder was homogeneously dispersed in deionized water, Darvan C, hydroxypropyl methylcellulose and polyethylenimine. The scaffolds were printed using a conical nozzle (410 μm) of an ink-filled syringe at 5 mm/s speed and strut spacing and layer height were maintained at 900 and 322 μm, respectively. After printing, the samples were air-dried for 24 h at room temperature, then heat treated at 400 °C for 1 h (binder burn-out), followed by sintering at 1200 °C for 1 h in a conventional furnace.

PLGA/TCP scaffolds were fabricated by using an LTRP machine (Tissue Form II, Tsinghua University, China). PLGA (lactide to glycolide molar ratio 3:1) and β-TCP powder were homogeneously mixed at a 4:1 weight ratio with 1,4-dioxane by overnight magnetic stirring at 37 °C and then loaded into the printing machine controlled robotically (Cark, Tsinghua University). The scaffolds with 322 μm layer height and 1.2 mm strut-spacing were printed at 20 mm/s speed through a 410 μm conical nozzle and subsequently freeze-dried (Christ Alpha 1-2 LD, U.K.).

All of the scaffolds were subsequently analyzed for surface topography and microstructure by scanning electron microscopy (SEM, S3400N, Hitachi, Japan) and microcomputed tomography (micro-CT, SkyScan1172, Bruker, Kontich, Belgium). Uniaxial compressive strengths of the fabricated scaffolds were also reported. For other biological studies, PLGA/TCP and TCP scaffolds were subsequently sterilized by γ radiation (Co^60^ source; 25 kGy) and autoclaving (120 °C, 1 h), respectively.

### 2.3 Degradation Assay

Both PLGA/TCP and TCP scaffolds (5 mm × 5 mm × 5 mm) were immersed in sterile Tris-HCl solution, pH 7.4 (1.25 mL/scaffolds) at 120 rpm, 37 °C (*n* = 10). At scheduled intervals (every week), the media was harvested for analyzing calcium and phosphate ion concentrations by ICPOES and immediately replaced with fresh medium after measurement of dry weight and dimensions of the scaffolds. Finally, the changes in scaffold morphology were analyzed under SEM.

### 2.4 Coating and rhBMP-2 Release Kinetics

Sterile PLGA/TCP and TCP scaffolds were coated with rhBMP-2 (from *E. coli*; purity >98%; Jiuyuan Genetic Institute of Huadong Medicine, China) for in vivo and in vitro release study. For in vivo study, 6 mg of rhBMP-2 was coated on each scaffold (20 mm × 10 mm × 10 mm). Briefly, 5 mL of rhBMP-2 solution (108 mg rhBMP-2 dissolved in 90 mL of 3% w/v gelatin solution prepared in 1% v/v acetic acid and the final rhBMP-2 concentration was 1.2 mg/mL) was added to each scaffold, then lyophilized for 24 h, and stored at 2–7 °C for further use. In vitro release kinetics of rhBMP-2 from the coated PLGA/TCP and TCP scaffolds (200 μg rhBMP-2 per 5 mm × 5 mm × 5 mm scaffold) was analyzed. For rhBMP-2 release study, the scaffolds (*n* = 3) were soaked in 1 mL of 10% phosphate buffer saline (PBS; pH 7.4, at 37 °C) in a closed centrifuge tube and agitated at 60 rpm. The total volume of the medium was collected after 3, 6, 9, 24, 48, 72, 120, 168, 336, and 504 h and thereafter analyzed by enzyme linked immunosorbent assay (ELISA) using a BMP-2 Elisa kit (Peprotech, U.S.A.) to calculate the percentage of rhBMP-2 release by dividing the measured contents (from a standard curve) with loaded amount of rhBMP-2. An equal volume of fresh medium was replaced every time after withdrawal of medium.

### 2.5 Cytocompatibility and Cell Proliferation Assay of Mandibular Scaffolds

Sterile scaffolds (5 mm × 5 mm × 5 mm) were seeded with human bone marrow mesenchymal stem cells (hBMSCs; 2.5 × 10^5^/scaffold) and cultured with Dulbecco’s modified eagle medium (DMEM, Gibco, U.S.A.) containing 10% fetal bovine serum (FBS, Gibco, U.S.A.) and 1% antibiotics (10 000 U/mL penicillin G and 25 μg/mL amphotericin B) in a humidified CO_2_ incubator at 37 °C. Cell proliferation was evaluated by Alama Blue kit (Invitrogen) on days 2, 4, and 6 post cells seeding. Cytoskeletal staining was also carried out to analyze cell morphology onto the scaffolds. Briefly, hBMSCs (2.5 × 10^5^/scaffold) were seeded onto the scaffolds and cultured for 3 d. The samples were fixed with 4% paraformaldehyde, penetrated with 0.1% Triton X-100, and finally incubated with rhodamine/phalloidin-labeled FITC solution (Cytoskeleton, U.S.A.) for 30 min followed by DAPI solution for 10 min at room temperature. The samples were carefully washed with PBS and observed under laser scanning confocal microscopy (CLSM, Leica).

### 2.6 Prefabrication of Tissue-Engineered Bone in Primate

Prefabrication and mandibular reconstruction surgeries were performed in nine healthy male rhesus monkeys (6–9 yrs., 6–12 kg) provided by Xusheng Biotechnology, Guangzhou, China and kept in SS cages (85 cm × 92 cm × 100 cm) at 25–27 °C under 12 h light-dark cycle. The study protocol was approved by Institutional Animal Care and Use Committee of Guangzhou Medical University, Guangzhou, China (2015–016). The animal handling procedure was according to the standard operating procedure of Experimental Animal Center of Nanfang Hospital, Guangzhou. Owing to the highly expensive and tedious procedure as well as the stringent regulatory control and ethical concerns associated with large animal study, a minimum number of animals (*n* = 3) was used to comply with the statistical significance of the obtained data. All surgeries were performed under general anesthesia with tracheal intubation based on a published protocol.^5^ General anesthesia was induced with ketamine (20 mg/kg) and maintained with 1% pentobarbital. Postoperative tramadol (50–100 mg/per monkey, i.m.) was given to alleviate the pain. Briefly, TCP (P-TCP, *n* = 3), rhBMP-2 coated TCP (P-TCP-BMP, *n* = 3), PLGA/TCP (P-PLGA/TCP, *n* = 3), and rhBMP-2 coated PLGA/TCP (P-PLGA/TCP-BMP, *n* = 3) scaffolds were individually loaded within customized titanium meshes and implanted bilaterally in ambilateral latissimus dorsi muscle. After implantation, wounds were immediately closed using absorbable sutures. Twelve weeks later, ossification was experimentally validated and based on the obtained data, prefabricated bone flaps were selectively transplanted to the actual segmental mandibular defects as per the following protocol.

### 2.7 Mandibular Reconstruction in Primate

Three months’ post teeth extraction, 20 mm long segmental defects (20 mm × 15 mm × 10 mm or approximately 3 cm^3^) were created by exposing mandible extra-orally through full-thickness periosteal flap, as planned in the virtual surgery in bilateral regions from the first premolar to third molar. PTEBs derived from P-TCP-BMP scaffolds along with the pedicled latissimus dorsi muscle flap containing uninterrupted arteriovenous blood supply were transferred to the mandibular defects via a tunnel created beneath the major pectoral muscle, namely P-TCP-BMP bone flap. Other orthotopic restorations were done by nonprefabricated TCP (S-TCP, *n* = 3), PLGA/TCP (S-PLGA/TCP, *n* = 3), rhBMP-2 coated TCP (S-TCP-BMP, *n* = 3), and rhBMP-2 coated PLGA/TCP (S-PLGA/TCP-BMP, *n* = 3) scaffolds, which were secured by titanium mesh and screws (2 mm diameter; Cixi, China) to the stumps of mandibular defects. All animals were maintained on a soft diet. A total of 2 g of cefradine i.v. injection was given twice daily for 7 days postoperatively. The ossification level was regularly assessed by clinical and radiographic examinations. Then 3 months after the mandibular reconstruction surgery, the animals were sacrificed with an overdose of pentobarbital sodium.

### 2.8 PET/CT Analysis

PET/CT imaging was performed at 4-, 8-, and 12-weeks’ postimplantation. The animals were positioned within PET/CT scanner (Discovery PET/CT Elite, General Electric Medical Systems, Milwaukee, WI, U.S.A.) 60 min after intravenous injection of 0.10–0.18 mCi/kg ^18^F-FDG. PET/CT images were acquired at 120 keV voltage, 120 mA current, 0.813 pitch, and reconstructed at 1.35 mm slice thickness using MedEx (MedEx, Beijing, China). The volume of interest was outlined within titanium mesh implanted in LDM and mandibular defects. Standardized uptake values (SUV) were calculated at a ratio of reconstructed scaffold volume in either LDM (SUV_L/V_) or mandibular defects (SUV_M/V_) to vertebra at the same axial section.

### 2.9 Angiographic and Radiographic Examination

Angiography was performed 2 h before sacrificing the animals in order to validate blood supply through thoracodorsal vascular bundle to the PTEB. Under general anesthesia, the femoral artery was exposed and dissected for cannulating the mesial and distal ends with glass tubes. After allowing 50 mL of blood outflow from the distal end of the artery, perfusate consisting of 0.9% NaCl solution, 30% gelatin, and 30% barium sulfate was injected at a dose of 20 mL/kg by the mesial end. Successful perfusion was ensured by the appearance of perfusate color in the oral mucosa as well as eyelid. Finally, the animals were sacrificed and kept at 4 °C overnight. The mandible specimens containing implanted scaffolds were harvested and subjected to X-ray radiographic examination (LWX-50P, Lanyun, China) for evaluating neo-bone formation and osseointegration between scaffolds and mandible.

In addition, ectopic and orthotopic bone formations within scaffolds were qualitatively and quantitatively evaluated by high-resolution micro-CT imaging with a resolution of 15 μm, using 80 kV and 100 μA radiation source fitted with a Cu Al filter. Volumetric reconstruction and analysis were performed using NRecon 1.1, CTan 1.13 and CTvol 2.0 (Bruker) software. The ratio of bone volume to total volume (BV/TV) was calculated. At least three samples were analyzed for each analysis.

### 2.10 Biomechanical Analysis

Biomechanical properties of the original, retrieved prefabricated as well as in situ implanted PLGA/TCP and TCP scaffolds were evaluated by uniaxial compressive testing. Before testing, specimens were reduced to a specific dimension of 4 mm × 4 mm × 4 mm and placed on a biomechanical testing machine (Instron, U.S.A.). All tests were conducted at 0.5 mm/min crosshead speed, and the compressive strength (MPa) was calculated by dividing the maximum applied load with initial cross-sectional area of the sample.

### 2.11 Histological Analysis

For histological analysis, the specimens were initially fixed with 4% paraformaldehyde for 48 h and dehydrated with graded ethanol solutions. Poly(methyl methacrylate) (PMMA) blocks were prepared by infiltrating PMMA solution for 2 weeks, and 200 μm slices were obtained by microtomy (Norderstedt, Germany). The thickness of each slice was reduced to 50 μm by polishing with Exakt grinder system (Norderstedt, Germany). Hematoxylin and eosin (H&E) staining was used for observation. Five random fields were captured by an optical microscope from three representative slides of each specimen. Percentage of bone volume (BV) and residual scaffold (RS) were calculated using Image-Pro Plus, version 6.0 (Media Cybernetics, Silver Spring, MD, U.S.A.).

### 2.12 Statistical Analysis

The obtained results were statistically analyzed by using SPSS 20.0 version (IBM Corp, Armonk, NY, U.S.A.), and data were represented as mean ± standard deviation. Statistical differences among various experimental groups were analyzed by one-way ANOVA. Post multiple comparisons were performed using least significant difference (LSD) test (assuming equal variance) or Tamhane’s test (assuming unequal variance). The significance level was set at *p* < 0.05.

## 3 Results

### 3.1 Fabrication of PLGA/TCP and TCP Scaffolds

**Figure 1:**
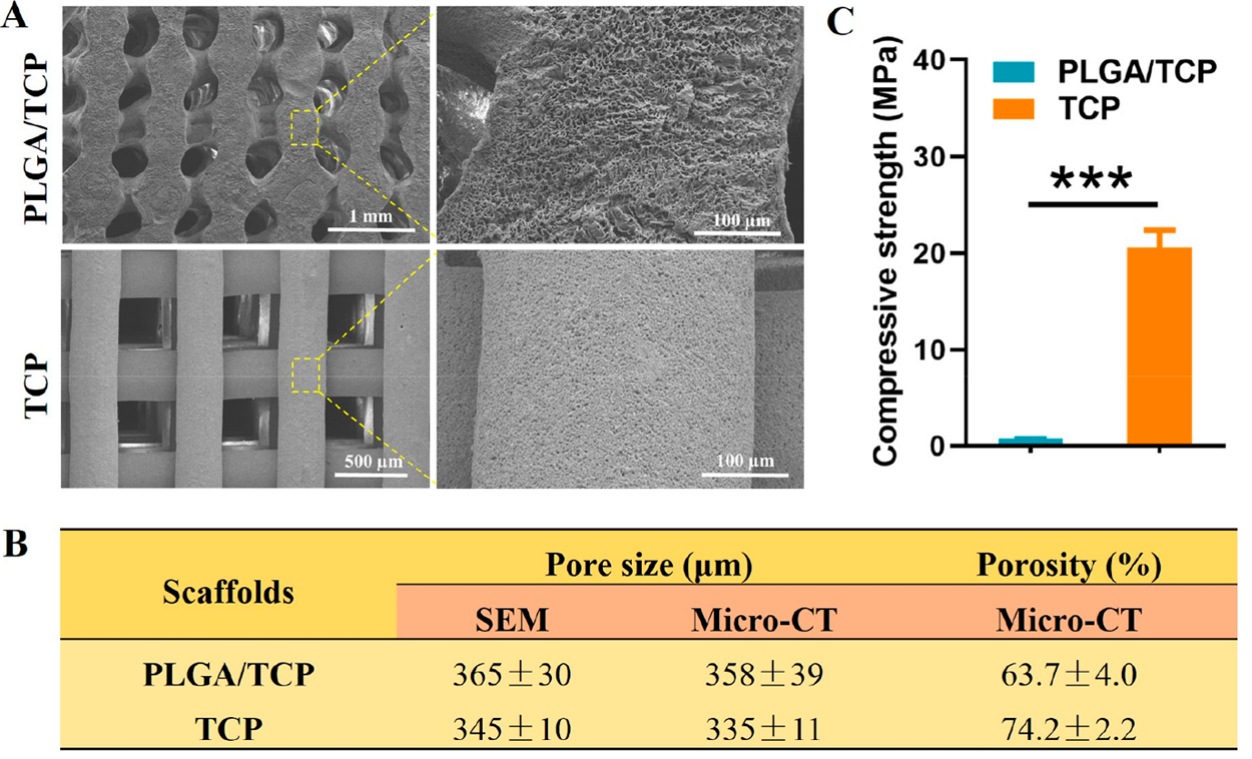
Morphological and mechanical characterization of PLGA/TCP and TCP scaffolds. (A) SEM micrographs; (B) pore size and porosity measured by SEM and micro-CT; (C) plot representing the data obtained from uniaxial compressive strength measurement of the scaffolds (*n* = 10, ***p < 0.05).

PLGA/TCP and TCP scaffolds were successfully fabricated by LTRP and robocasting, respectively. Under SEM, TCP scaffolds displayed a regular porous structure with a pore size of 345 ± 10 μm (Figure 1A,B). PLGA/TCP scaffolds also exhibited a structurally similar porous structure with pore size of 365 ± 30 μm. However, compared to PLGA/TCP the surface of TCP is more regular and even smoother. In micro-CT analysis, the pore sizes were measured to be 358 ± 39 and 335 ± 11 μm, respectively. However, the porosity of PLGA/TCP scaffolds was lower (63.7% ± 4.0%) than that of TCP scaffolds (74.2% ± 2.2%). In addition, the measured compressive strengths of PLGA/TCP scaffolds were dramatically lower (0.7 ± 0.06 MPa) than that of TCP (20.56 ± 1.81 MPa; Figure 1C).

### 3.2 Degradation Assay

**Figure 2:**
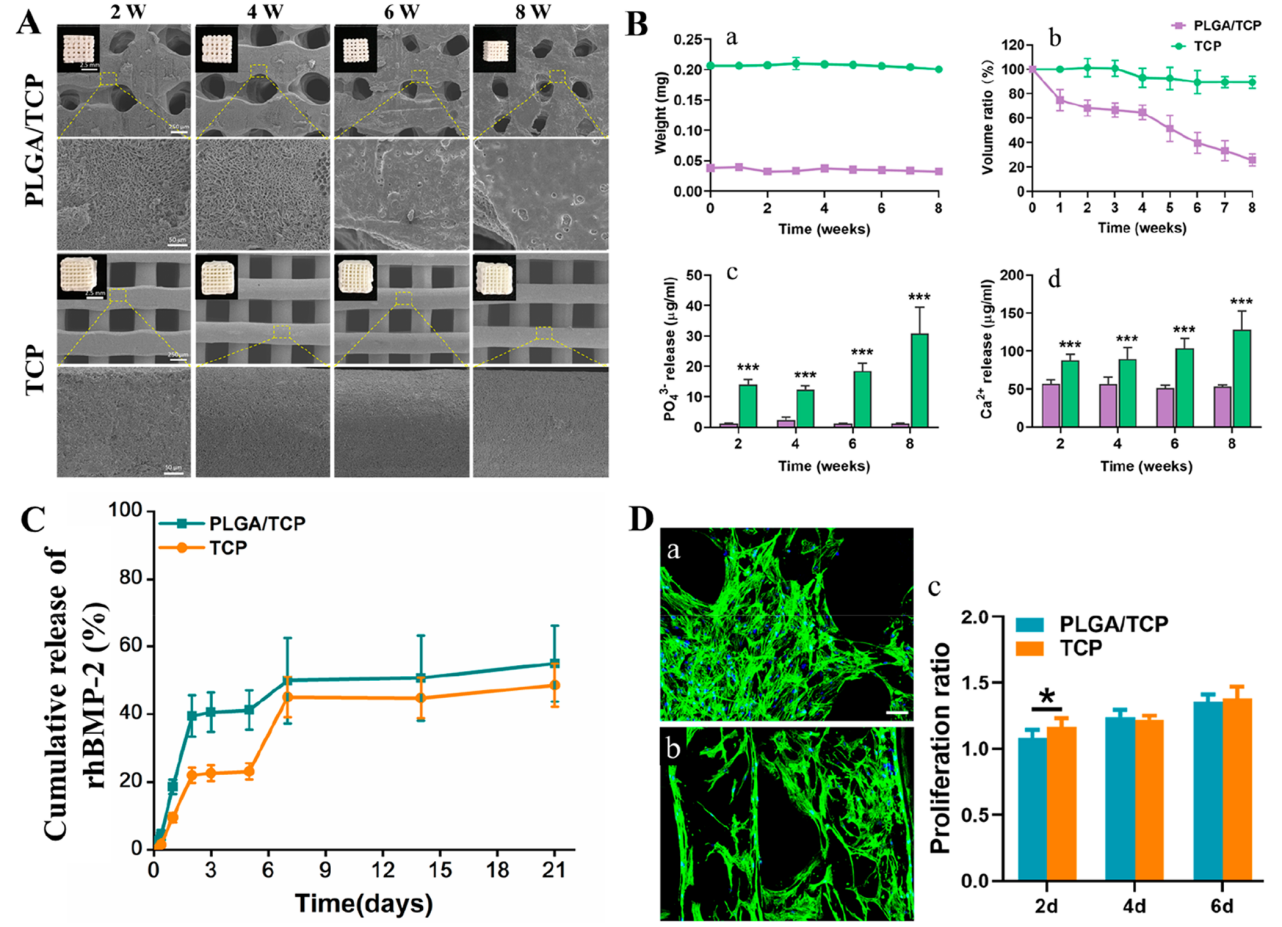
(A) Gross and magnified view of PLGA/TCP and TCP scaffolds under SEM retrieved after 2, 4, 6, and 8 weeks of degradation study (inset: optical images of the scaffolds); scale bar: same for all time points as shown in the first image of each series. (B) Changes in scaffold weight and volume during degradation study (a, b) and ICP-OES elemental analysis of the supernatant obtained after scaffolds degradation (c, d). (C) rhBMP-2 release kinetics from rhBMP-2 coated PLGA/TCP and TCP scaffolds. (D) Fluorescence imaging of cultured hBMSCs after cytoskeletal staining using rhodamine/phalloidin-labeled FITC and nuclear staining of DAPI on (a) PLGA/TCP and (b) TCP scaffolds surface. Scale bar = 100 μm; (c) cell proliferation rate after 2, 4, and 6 days of cell seeding.

Significant changes (steady shrinkage of scaffold volume and disappearance of microporous structure) were observed for PLGA/TCP scaffolds at 2, 4, 6, and 8 weeks of degradation study (Figure 2A). Since PLGA/TCP scaffolds have a soft polymeric base which undergoes structural changes similar to shrinkage, the pores of the PLGA/TCP scaffolds appeared to be smaller than earlier time points. On the contrary, TCP scaffolds exhibited almost no morphological changes. In general, both PLGA/TCP (0.038 ± 0.005 vs 0.032 ± 0.005 g) and TCP (0.206 ± 0.004 vs 0.200 ± 0.004 g) did not show obvious weight loss over the time course of the study (Figure 2B,a). However, volumetric shrinkage of the PLGA/TCP scaffolds was estimated to be 25.6% ± 4.9% of the original size, whereas TCP scaffolds were able to retain 89.5% ± 4.9% of its original volume up to 2 months (Figure 2B,b). Moreover, both Ca^2+^ and PO_4_^3–^ ion concentrations in the supernatant obtained from TCP scaffolds were significantly higher (∼1.7–1.9 and ∼11.8–27.1 fold, respectively) than that of the PLGA/TCP scaffolds (Figure 2B,c,d). In turn, TCP scaffolds were expected to have a higher tendency to induce localized mineralization.

### 3.3 rhBMP-2 Release Kinetics

rhBMP-2 release kinetics from PLGA/TCP and TCP scaffolds is displayed in Figure 2C. For both scaffolds, active rhBMP-2 release was found to be linear in the first 2 days followed by periodic release in a sustained manner from day 3 to day 21. Due to higher porosity and pure crystalline calcium phosphate (CaP) structure, there was a strong binding of deeper penetrated rhBMP-2 within TCP scaffolds, which ultimately retarded rhBMP-2 release. On the other hand, faster release of rhBMP-2 was mainly from PLGA/TCP scaffold surface exposed to the release medium. This was also evidenced from SEM images that the incorporated rhBMP-2 was homogeneously distributed throughout the porous TCP scaffolds compared to limited distribution on outer surface of PLGA/TCP scaffolds (Figure S2). The cumulative release of rhBMP-2 from PLGA/TCP and TCP scaffolds were found to be 54.9% ± 11.2% and 48.5% ± 6.4% of the loaded rhBMP-2, respectively after 21 days of release study.

### 3.4 Cytocompatibility and Cell Proliferation Assay of Mandibular Scaffolds

Biocompatibility of PLGA/TCP and TCP scaffolds was determined by culturing hBMSCs on the scaffolds. Apart from formation of a polygonal morphology, the cells were rapidly proliferated along the struts of PLGA/TCP and TCP scaffolds and radially migrated across the pores (Figure 2D,a,b). From the data plot in Figure 2D,c, there was a significant difference in cell viability on day 2 of cell seeding between PLGA/TCP and TCP scaffolds; however, the difference became insignificant after day 4, indicating their cytocompatible nature.

### 3.5 Prefabrication of Tissue-Engineered Bone in Primate

The monkeys remained healthy after the surgical trauma without any necrotic symptoms of the muscle tissue. However, local swelling with rhBMP-2 coated scaffolds lasted longer than uncoated scaffolds. Twelve weeks’ postimplantation, the original shape of PLGA/TCP scaffolds with/without rhBMP-2 was significantly changed, turning to a muddy appearance (Figure S5a,b). P-PLGA/TCP-BMP scaffolds showed less bone formation on part of their surfaces, thereby they were excluded from further study. However, pure TCP scaffolds with/without BMP-2 coating could retain their original shapes (Figure S5c,d), and an even layer of bone was formed around rhBMP-2 coated TCP scaffolds (Figure S5d), which was later transferred with pedicle to restore critical-sized mandibular defect.

### 3.6 Mandibular Reconstruction in Primate

**Figure 3:**
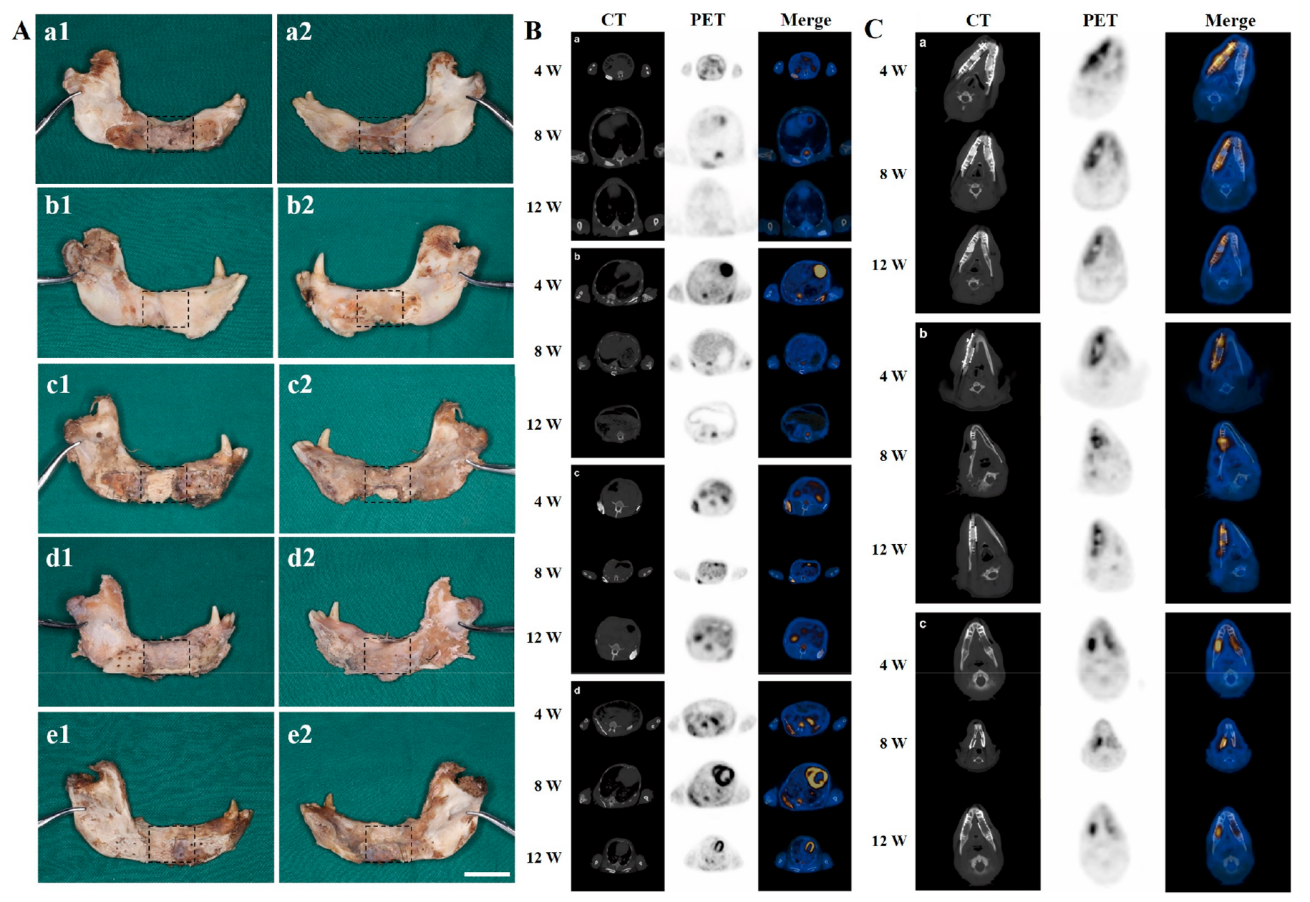
(A) Gross views of specimens retrieved after 12 weeks of orthotopic implantation using (a) S-PLGA/TCP, (b) S-PLGA/TCP-BMP, (c) S-TCP, (d) S-TCP-BMP, and (e) P-TCP-BMP bone flap constructs (1 and 2 refer to the buccal and lingual sides, respectively; scale bar = 20 mm). (B and C) PET/CT imaging after 4-, 8-, and 12-weeks’ postimplantation of (B) TCP (a), PLGA/TCP (b), TCP-BMP (c), and PLGA/TCP-BMP (d) scaffolds in LDM and (C) P-TCP-BMP bone flap (a, left), TCP (a, right), TCP-BMP (b), PLGA/TCP-BMP (c, left), and PLGA/TCP (c, right) scaffolds in mandibular defects.

Healing of mandibular defects was almost incomplete using prefabricated S-PLGA/TCP, S-PLGA/TCP-BMP and S-TCP scaffolds implanted orthotopically (Figure 3A,a–c). However, S-TCP-BMP and P-TCP-BMP bone flap groups successfully restored the defect region (Figure 3A,d,e). Adhered muscle flaps along with the harvested P-TCP-BMP bone flap retained within the transplantation site for more than 12 weeks, and such scaffolds led to regeneration of a higher bone volume and almost reconstruct mandibular shape and integrity.

### 3.7 PET/CT Analysis

The data obtained through PET/CT scanning at 4-, 8- and 12-weeks’ postimplantation in ectopic and orthotopic sites are presented in Figure 3B,C, respectively. The scanning parameters used for PET/CT analysis could not reveal details of bone ingrowth into the porous structure of the scaffolds. However, density values obtained from TCP scaffolds (in Figure S3) were found to be higher than PLGA/TCP scaffolds due to higher calcium contents. The values persistently increased for TCP groups, whereas PLGA/TCP scaffolds (with/without rhBMP-2 coating) showed a decrease over the time for both ectopic and orthotopic implantation. SUV_L/v_ and SUV_M/v_ values representing ^18^F-FDG uptake are plotted in Figure S4. Overall, ^18^F-FDG uptake for the scaffolds without rhBMP-2 coating decreased over time, while PLGA/TCP scaffolds exhibited higher ^18^F-FDG uptake compared to that for the TCP scaffolds, irrespective of ectopic or orthotopic implantation. Coating of rhBMP-2 significantly enhanced the density values over time, especially at 8 weeks postimplantation. Nevertheless, the implantation site had significant influence on ^18^F-FDG uptake and density value in the case of rhBMP-2 coated scaffolds (higher uptake at mandibular site compared to muscle), though such a difference was absent in rhBMP-2 uncoated scaffolds. Density values for PLGA/TCP scaffolds placed at orthotopic sites were comparatively lower than those that were placed at ectopic sites, while pure TCP scaffolds did not show any such differences. Here it should be mentioned that the prefabricated P-TCP-BMP bone flap constructs resulted in lower ^18^F-FDG uptake and higher density values compared to that of directly implanted S-TCP-BMP scaffold groups.

### 3.8 Angiographic and Radiographic Examination

**Figure 4:**
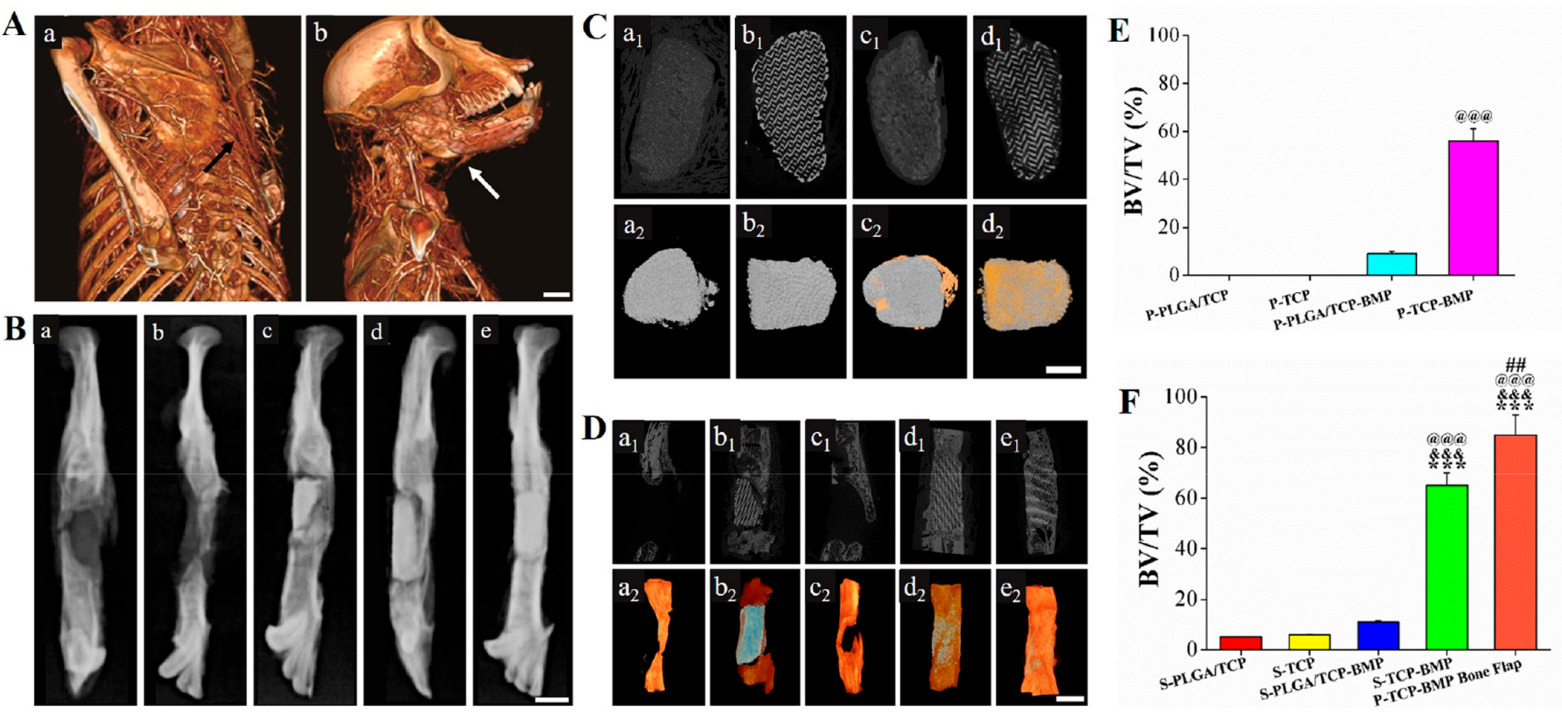
(A) Angiography of tissue-engineered grafts endocultivated in LDM and supplied with thoracodorsal vessels (scale bar = 20 mm). (B) Radiography of scaffolds after 3 months of orthotopic implantation. (a) S-PLGA/TCP, (b) S-PLGA/TCP-BMP, (c) S-TCP, (d) S-TCP-BMP, and (e) P-TCP-BMP bone flaps. P-TCP-BMP bone flaps exhibiting favorable osseointegration, while the others left detectable radiolucent gaps at bone-implants interface (scale bar = 10 mm). micro-CT images of (C) ectopically (scale bar = 5 mm) and (D) orthotopically implanted scaffolds (scale bar = 10 mm): (a) PLGA/TCP, (b) TCP, (c) PLGA/TCP-BMP, (d) TCP-BMP, and (e) P-TCP-BMP bone flaps (1 and 2 refer to 2D and 3D reconstruction, respectively). Blue and gray: scaffolds; yellow: new bone. Percentage of bone volume (BV) to total tissue volume (TV) calculated for (E) ectopically and (F) orthotopically implanted scaffolds. Significant (*p* < 0.001) increase over *** S-PLGA/TCP, ^&&&^ S-TCP, ^@@@^ PLGA/TCP-BMP, and (*p* < 0.01) ^##^ S-TCP-BMP.

Angiography was performed to assess pedicled bone flap viability along with the state of blood supply. The prefabricated constructs were surrounded by copious branches and networks of newly grown thoracodorsal blood vessels (Figure 4A,a). After transplantation, the thoracodorsal bundles were clearly visible between axillary fossa and inferior margin of the reconstructed mandibular defects (Figure 4A,b), and they also seemed to be one of the major sources of blood supply to the transplanted tissue-engineered construct.

Radiographic examination was performed to detect the level of osseointegration between scaffolds and the mandible. The radiopaque areas within mandibular defects containing S-TCP, S-TCP-BMP, and P-TCP-BMP bone flaps were comparatively higher than those with S-PLGA/TCP and S-PLGA/TCP-BMP (Figure 4B,a–e), indicating the failure of bone regeneration in S-PLGA/TCP and S-PLGA/TCP-BMP scaffold groups with less residual materials (Figure 4B,a,b). However, mandibular discontinuity was successfully repaired using S-TCP-BMP and P-TCP-BMP bone flap groups, which displayed a higher radiopacity value (Figure 4B,d,e). In addition, bone overgrowth was more obvious in S-TCP-BMP group than P-TCP-BMP bone flap group. A clear visibility of the distal part between scaffold material and host bone as well as low radiopacity value resulting from less bone content indicated failure of osseointegration between implanted S-TCP scaffolds with host bone (Figure 4B,c).

From micro-CT images in Figure 4C, the ectopically implanted P-PLGA/TCP and P-PLGA/TCP-BMP scaffolds underwent significant degradation, with reduced scaffold density and distorted shape as well as inner structure. Bone formation was insignificant in the former case (Figure 4C,a), while the latter induced comparatively higher bone formation in the periphery along with a layer of soft tissue (Figure 4C,c). Both P-TCP-BMP and P-TCP scaffolds could retain their original shape and internal structure, besides formation of a uniform bone layer around P-TCP-BMP scaffolds (Figure 4C,b,d). Quantitative analysis showed that bone density and BV/TV values were significantly higher (*p* < 0.05) of P-TCP-BMP scaffolds compared to the other groups (Figure 4E).

In the case of orthotopic implantation, S-PLGA/TCP and S-PLGA/TCP-BMP scaffolds were significantly resorbed after 12 weeks of implantation and the scaffolds were almost invisible in the reconstructed images (Figure 4D,a,c). Remarkably, higher bone regeneration was evident in mandibular defects repaired with S-PLGA/TCP-BMP than that of S-PLGA/TCP. Inner porous structure of S-TCP and S-TCP-BMP scaffolds remained nearly the same even after 3 months of implantation, while the exterior part was partly integrated to the host bone (Figure 4D,b,d). Only S-TCP-BMP and P-TCP-BMP bone flap groups could successfully repair the mandibular defects through neo-bone formation (Figure 4D,d,e). Moreover, the transplanted P-TCP-BMP bone flap could form mature bone (high BV/TV ratio) into the hardly visible blue-colored scaffolds restoring the original shape of the mandible. This clearly indicated a promising strategy for accelerated recovery of large mandibular defects (Figure 4F).

### 3.9 Biomechanical Analysis

**Figure 5:**
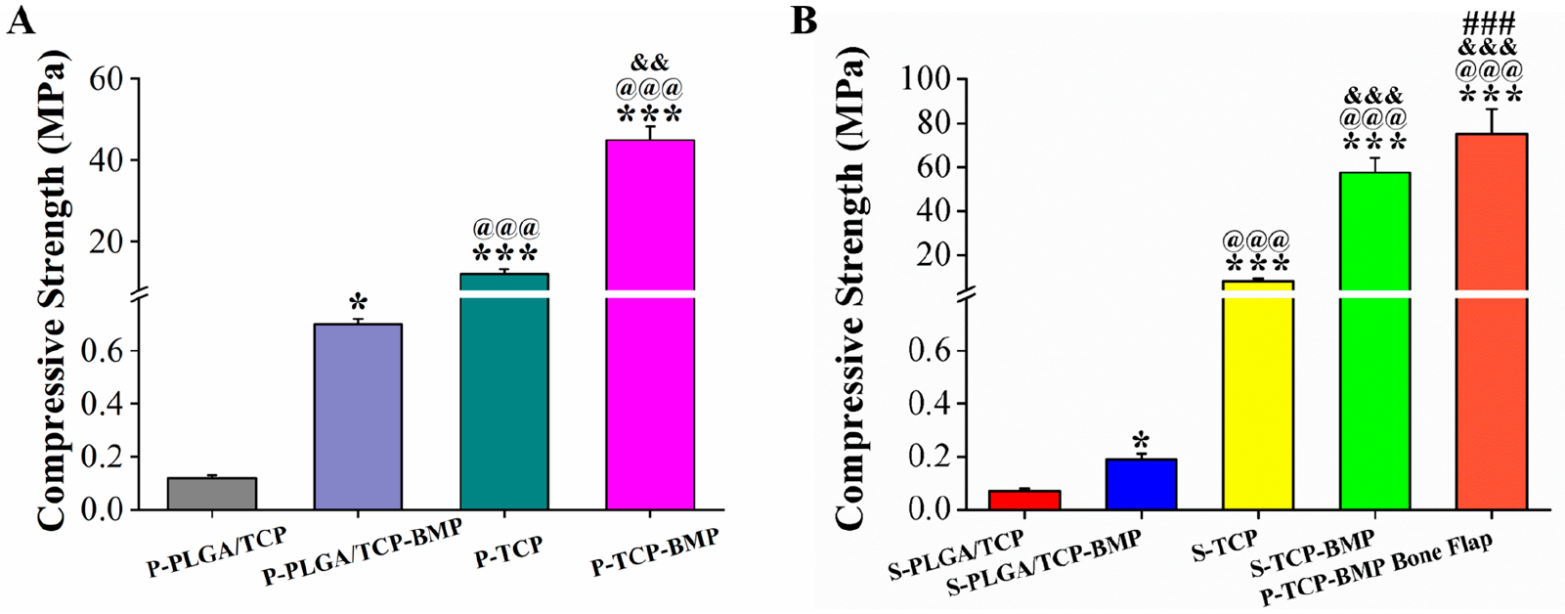
Compressive strengths of 3D printed scaffolds after 3 months of (A) endocultivation (significant increase over P-PLGA/TCP (* *p* < 0.05, *** *p* < 0.001), ^@@@^ P-PLGA/TCP-BMP (*p*^<^ 0.001), ^&&^ P-TCP (*p* < 0.01)) and (B) orthotopic implantation (significant increase over S-PLGA/TCP (*p < 0.05, ***p < 0.001); ^@@@^ S-PLGA/TCP-BMP (*p* < 0.001), ^&&&^ S-TCP (*p* < 0.001), ^###^ S-TCP-BMP (*p* < 0.001).

The mechanical strengths of composite P-PLGA/TCP (0.1 ± 0.01 MPa) and P-PLGA/TCP-BMP (0.7 ± 0.02 MPa) scaffolds were significantly reduced (*p* < 0.001) compared to P-TCP and P-TCP-BMP scaffolds after prefabrication, while P-TCP-BMP scaffolds (45.0 ± 3.3 MPa) exhibited more than 3-fold strength (*p* < 0.01) compared to P-TCP scaffolds (12.0 ± 1.3 MPa; Figure 5A). Similar strength reduction was also found with orthotopically implanted S-PLGA/TCP and S-PLGA/TCP-BMP scaffolds. However, S-PLGA/TCP-BMP scaffolds exhibited comparatively higher strength (0.2 ± 0.02 MPa; *p* < 0.05) than that of S-PLGA/TCP scaffolds (0.1 ± 0.01 MPa) due to rhBMP-2 guided bone regeneration. Similar effects were found with S-TCP-BMP scaffolds (57.6 ± 6.7 MPa) over their bare S-TCP counterparts (8.4 ± 1.2 MPa). Nonetheless, the highest strength was obtained with P-TCP-BMP bone flap group (75.2 ± 11.1 MPa) due to remarkably higher extent of bone formation (Figure 5B).

### 3.10 Histological Analysis

**Figure 6:**
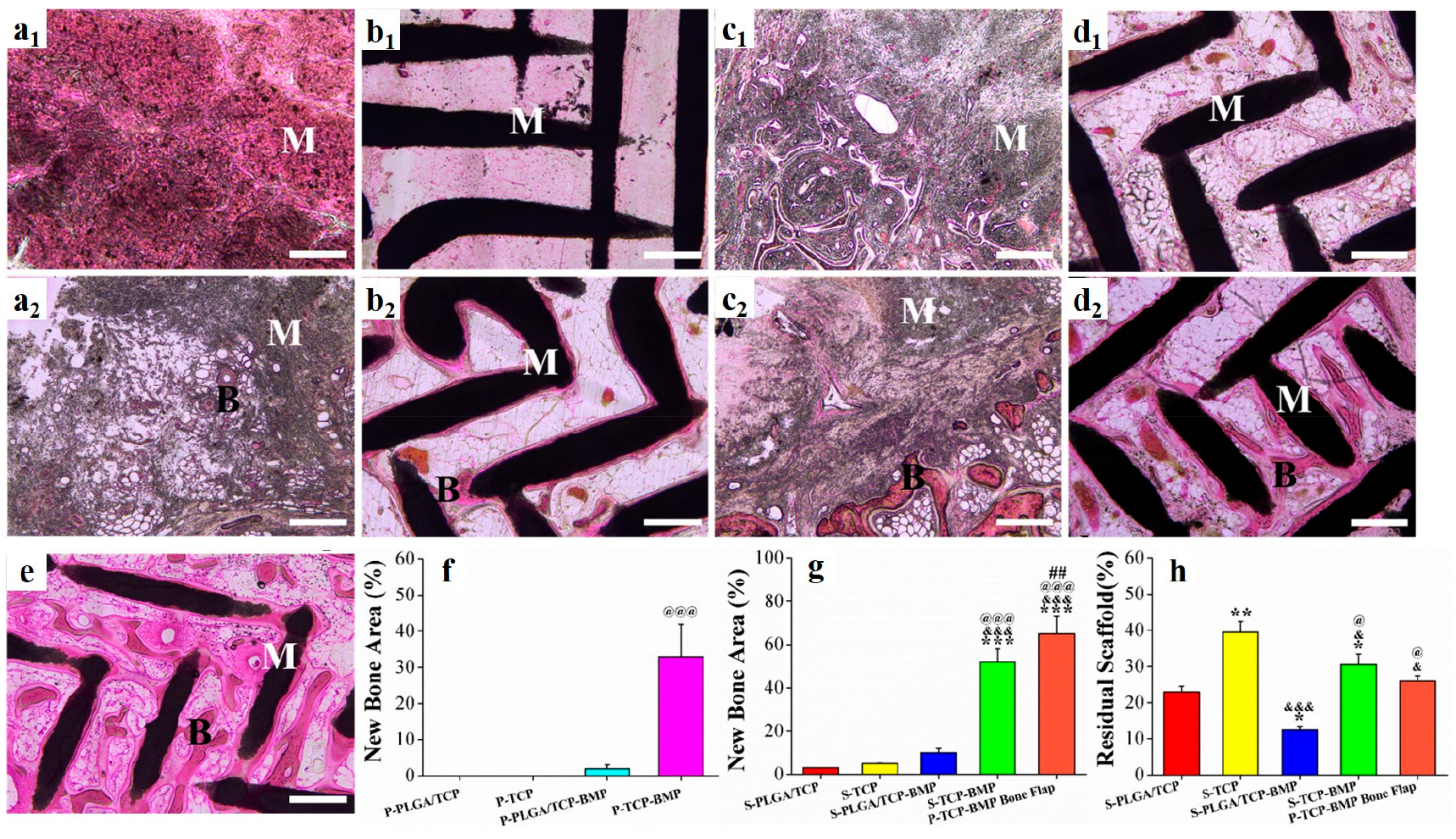
Representative images after H&E staining of samples retrieved after 12 weeks of implantation with (a) P-PLGA/TCP, (b) P-TCP, (c) S-PLGA/TCP, (d) S-TCP, and (e) P-TCP-BMP bone flap scaffolds (1 and 2 represents scaffolds without or with rhBMP-2 coating, respectively). M = materials, B = bone. Percentage of new bone in histological sections obtained from (f) ectopically (significant increase ^@@@^ over P-PLGA/TCP-BMP (*p* < 0.001)) and (g) orthotopically implanted specimens (significant increase over *** S-PLGA/TCP (*p* < 0.001), ^&&&^ S-TCP (*p* < 0.001), ^@@@^ S-PLGA/TCP-BMP (*p* < 0.001), ^##^ S-TCP-BMP (*p* < 0.01). (h) Percentage of nondegraded scaffolds in different specimens (significant increase over S-PLGA/TCP (*p < 0.01 and **p < 0.05), S-TCP (^&&&^*p* < 0.001 and ^&^*p* < 0.05) and S-PLGA/TCP-BMP (^@^*p* < 0.05). Scale bar = 400 μm.

H&E staining of the retrieved P-PLGA/TCP and P-PLGA/TCP-BMP constructs after 12 weeks of implantation in LDM clearly showed soft callus formation along with low bone density at the periphery of P-PLGA/TCP-BMP scaffolds (Figure 6a2). Although P-TCP scaffolds were able to retain their original uniform porous structure due to minimum degradation, they were mostly infiltrated with fibrous tissue and blood vessels (Figure 6b1). In contrast, surface of P-TCP-BMP scaffolds were covered with an uneven layer of bone (Figure 6b2). At orthotopic sites, S-TCP-BMP and P-TCP-BMP bone flap scaffolds were occupied with a large number of regenerated bone lacuna osseointegrated with the host bone (Figure 6d2,e). The S-TCP-BMP scaffolds displayed a large amount of residual β-TCP material with more new bone formation along the residual struts (Figure 6d2). P-TCP-BMP bone flaps exhibited the largest amount of bone regeneration (*p* < 0.05; Figure 6e). The dark gray regions in the histology images in Figure 6a1,a2,c1,c2, did not show any cellular structure associated with tissue necrosis or appearance of macrophages/other inflammatory cells. The ratio of bone volume to scaffold volume was calculated by ImageJ software from multiple histological images after H&E staining of the sections obtained from different locations of the same sample. The distinctive black (due to very high concentration of CaP) and dark pink colors (due to low concentration of CaP) obtained through H&E staining of scaffold material and newly grown bony tissue, respectively, were quantified by ImageJ. The semiquantitative data for new bone formation was in accord with the micro-CT data (Figure 6f–g). There was almost no new bone formation after ectopic implantation of PLGA/TCP and TCP scaffolds, whereas rhBMP-2 coated TCP scaffolds formed significantly higher amount of bone compared to rhBMP-2 coated PLGA/TCP (Figure 6f). Regarding orthotopic implantation, the scaffolds virtually followed a similar trend; however, the prefabricated rhBMP2-coated TCP scaffolds along with the bone flap (P-TCP-BMP bone flap) led to a significantly higher amount of bone formation compared to the direct implantation of rhBMP2-coated TCP scaffolds at the orthotopic site (S-TCP-BMP). This clearly indicated that PTEB based on rhBMP2-coated TCP scaffolds is a highly effective technique for faster recovery of bone defects. Besides, such TCP scaffolds (both prefabricated and nonprefabricated) could maintain their original structure, while the PLGA/TCP scaffolds were remarkably resorbed (Figure 6h).

## 4 Discussion

Among the highly explored biomaterials in orthopedics,^29−31^ PLGA/TCP and TCP revealed excellent biocompatibility and osteoconductivity while repairing bone defects in animal as well as human models.^14,17,18,28^ Also, the advent of 3D printing techniques has enabled us to customize PLGA/TCP and TCP scaffolds and to investigate their ability for reconstructing large mandibular defects in primate. Undeniably, an implantation study in a primate model has more clinical relevance to human applications. The in vitro osteogenic properties of both PLGA/TCP and TCP scaffolds were already evaluated in our earlier studies.^32,33^ 3D-printed PLGA/TCP could be a promising carrier of rhBMP-2 to induce ectopic bone formation in rat.^28^ Using rhBMP-2 coated 3D-printed TCP scaffolds, maxillary defects in minipigs were also successfully recovered.^14^ Based on earlier studies, PLGA/TCP and TCP were considered to be highly suitable candidates for prefabrication of large mandibular constructs with adequate vascularization. In the present study, both the scaffolds were able to maintain uniform pore sizes larger than 300 μm ensuring cellular activity, vascularization, along with the deposition of mineralized matrix.^34,35^

Prefabrication is a well-known surgical procedure to cultivate tissue-engineered bone or a large tissue-engineered construct within the host body parts (i.e., endocultivation in muscle or greater omentum) acting as an in vivo bioreactor.^36,37^ Prefabrication is necessary for those patients who underwent radiotherapy after ablative surgery of malignant tumors and thereafter suffered from lack of favorable blood supply and osteogenic environment within in situ defects. Vascularization of PTEB was found to be greatly improved by endocultivation technique due to the penetration of host blood vessels from the neighboring tissue, apart from the immigration of autologous stem cells from blood. Owing to the distal location from oral cavity and copious blood supply from the thoracodorsal artery, latissimus dorsi muscle (LDM) was chosen as a primary site for prefabrication. The in vivo working model and the study duration were selected based on our previous studies as well as the other contemporary works.^5,20^ On the basis of the contemporary articles, titanium meshes were custom-designed as a fixation device for additional mechanical support to the scaffolds during ectopic implantation, better immobilization of transplanted grafts into orthotopic sites, as well as prevention of bone overgrowth so that perfect fitting is possible to the mandibular defect. The meshes were fitted with flanges to immobilize the regenerated bone to the stump of mandible and holes to support vascular growth from the latissimus dorsi muscle. The height of mesh was less than that of the resected mandible to avoid its exposure to the oral cavity. The aforementioned strategy is comparatively new in clinical dentistry for reconstruction of complex defects in oral and maxillofacial region. Complications like osteolysis in mandibular contact regions surrounding the mesh was not observed until the end of this study. The previous studies also did not report any such incidences, since the masticating load was mainly taken up by the TCP graft itself (since titanium mesh remained outside) and successfully distributed to the other parts of the mandible.

From 2D sagittal views of micro-CT images, P-TCP-BMP bone flaps displayed better osseointegration and recovery of the mandibular defect due to its prevascularization as compared to other orthotopically implanted scaffolds. Bone overgrowth was much higher in the lingual side than in the buccal side in other orthotopically implanted scaffolds, similar to the previous reports.^38^ Buccal bone regeneration was partially hampered due to the damage of buccal periosteum during mandibular osteotomy, disrupting regular supply of pluripotent stem cells crucial for bone regeneration.^39^ The copious blood supply in LDM significantly made for faster degradation of PLGA/TCP composite scaffolds which shrank rapidly compare to TCP scaffolds. pH in the local microenvironment plays a crucial role for regulating osteogenic differentiation of mesenchymal stem cells and ectopic bone formation,^40^ although β-TCP, present in PLGA/TCP composite scaffolds, neutralized acidic degradation products of PLGA, i.e., lactic acid and glycolic acid. As a result, rhBMP-2 mediated ossification and other cellular activities were significantly affected through disruption of the native scaffold structure causing burst release of rhBMP-2. Since rhBMP-2 sensitivity relies on animal hierarchy,^41^ this could be a valid reason for better osteoinductivity of rhBMP-2 in rabbits compared to primates. After 12 weeks of prefabrication, compressive strengths of S-TCP-BMP and P-TCP-BMP bone flap (12.0 ± 1.3 MPa and 45.0 ± 3.3 MPa) were found between those of trabecular bone (2–12 MPa) and cortical bone (100–150 MPa).^42^ The ultimate compressive strength of the trabecular bone in the human mandible was reported to be 0.22–10.44 MPa.^43^ After prefabrication there was slight reduction of compressive strength for TCP scaffolds; however, there was a significant increase for TCP-BMP scaffolds. Notably P-TCP-BMP bone flap exhibited the highest compressive strength among all scaffold groups. Though S-TCP-BMP scaffolds exhibited superior mechanical properties (57.6 ± 6.7 MPa vs 45.0 ± 3.3 MPa, respectively) than prefabricated P-TCP-BMP scaffolds after 3 months of ectopic implantation, the strength of P-TCP-BMP (75.2 ± 11.1 MPa) surpassed S-TCP-BMP when transplanted to the orthotopic site due to continuous bone remodeling. Therefore, mechanical properties of bone construct could be enhanced by prefabrication. PLGA/TCP-BMP scaffolds displayed much lower strength due to faster degradation. The mechanical properties as well as composition of the scaffolds created significant differences in osteogenic activity and mineralization.

^18^F-fluoride labeled glucose uptake was successfully utilized for monitoring of BMP-2 activity.^44−46^^18^F-FDG uptake mainly depends on material type (polymer, ceramic or composite), scaffold degradation (biodegradable or nonbiodegradable), biological stimuli as well as the site of implantation. The present study utilized ^18^F-FDG as a tracer to indirectly determine effects of rhBMP-2 released from scaffold surface via assessment of metabolic activity (i.e., high ^18^F-FDG uptake due to high metabolic activity and high tissue turnover). The dose of ^18^F was calculated strictly based on monkey’s body weight according to the published reports. No behavioral changes or food intake abnormalities were observed after PET/CT scans.^47−49^ High metabolic activity was correlated with the faster degradation of PLGA/TCP scaffolds leading to a burst release of rhBMP-2, which, in turn, enhanced cellular activity and rapid resorption of the scaffolds. Therefore, ^18^F-FDG PET/CT could be explored as a novel noninvasive and indirect technique for real-time monitoring of in vivo bone regeneration. As expected, ^18^F-FDG uptake was greatly enhanced by incorporating rhBMP-2 in both scaffold types (PLGA/TCP-BMP and TCP-BMP), indicating higher bone formation in the TCP-BMP group. Notably, ^18^F-FDG uptake does not rely only on rhBMP-2 activity but is also influenced by inflammation, scaffold degradation, etc.^50,51^ Though rhBMP-2 induced higher ^18^F-FDG uptake in the PLGA/TCP composite, bone formation was not enhanced significantly, possibly due to the loss of the internal porous structure.^52^ Recently, various TCP based xenografts were clinically approved by the U.S. Food & Drug Administration (FDA), National Medical Product Administration (NMPA), and other regulatory bodies due to their advantageous properties: bioresorbable being analogous to bone apatite, excellent biocompatibility, and easy customization with intricate 3D designs of the inner structure. Moreover, prefabricated TCP scaffolds could maintain original shape and mechanical strength to withstand masticating loads. When combined with stem cells and growth factors, TCP scaffolds successfully recovered critical bone defects. In summary, 3D-printed TCP scaffolds showed much better in vivo stability, biomechanical properties, as well as osteoconductivity, thereby establishing its superiority over PLGA/TCP scaffolds. Considering the above aspects, prefabricated TCP-BMP bone flap tissue-engineered constructs can be indubitably better than conventionally implanted S-TCP-BMP scaffolds for the repair of large mandibular defects.

## 5 Conclusion

In summary, the present study compared the effects of prefabricated, tissue-engineered grafts on mandibular reconstruction, based on 3D printed PLGA/TCP and pure β-TCP scaffolds with/without rhBMP-2 coating. The major outcomes of the study include the unsuitability of PLGA/TCP composite scaffolds for prefabricating large tissue-engineered constructs due to faster degradation and subsequent loss of internal porous hierarchy. On the other hand, TCP scaffolds containing rhBMP-2 provided more predictable and consistent results in primates owing to their shape retention for a much longer period, adequate neo-vascularization, and mineralization. The study also investigated the potential of ^18^F-FDG PET/CT in monitoring the biological effects of rhBMP-2 released from the implanted scaffolds. Overall, the outcome of this study mainly emphasized the immense potential of prefabricated P-TCP-BMP bone flaps for the clinical restoration of large compound mandibular defects.

## Supporting Information Available

Schematic illustration of the study; SEM images of PLGA/TCP and TCP scaffolds coated with rhBMP-2; density values (Hounsfield units) of the scaffolds; ^18^F-FDG uptake for different scaffolds; gross views of ectopically implanted scaffolds after three months (PDF)
