# Supplementary material for: Prefabricated 3D-Printed Tissue-Engineered Bone for Mandibular Reconstruction: A Preclinical Translational Study in Primate
Source: ACS Biomater Sci Eng. 2021 Nov 22;7(12):5727–38. doi: 10.1021/acsbiomaterials.1c00509 (PMC8672350; doi:10.1021/acsbiomaterials.1c00509)

Supplementary Material

Prefabricated 3D-printed Tissue-engineered Bone for Mandibular Reconstruction: A Preclinical Translational Study in Primate

*Shuai-shuai Cao[1, #], Shu-yi Li[1, 2, #], Yuan-ming Geng[3, #], Kausik Kapat[1], Shang-bin Liu[1], Fidel Hugo Perera[4], Li Qian[5], Hendrik Terheyden[6], Gang Wu[7], Yue-juan Che[8, *], Pedro Miranda[4,*], Miao Zhou[1, *]*

S1. Schematic illustration of the study.

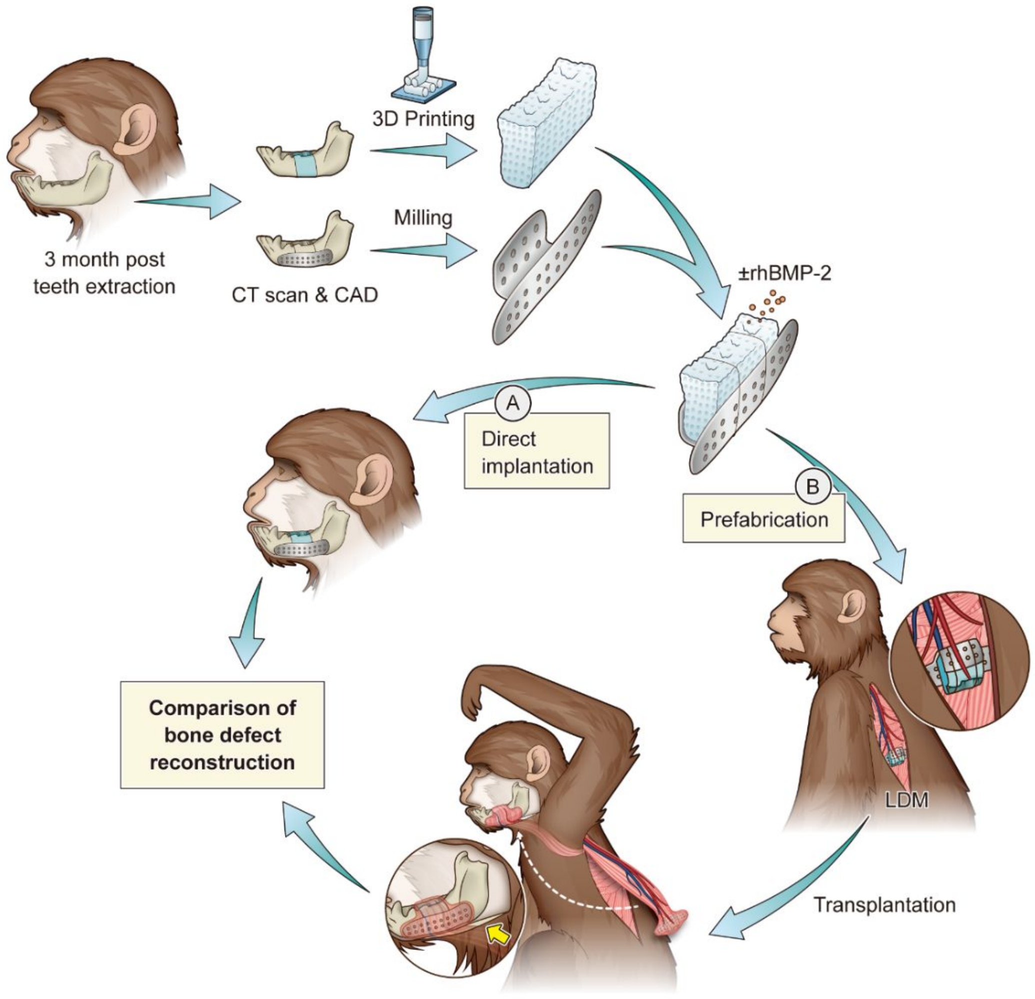

S2. SEM images of PLGA/TCP (a & b) and TCP (c & d) scaffolds coated with rhBMP-2.

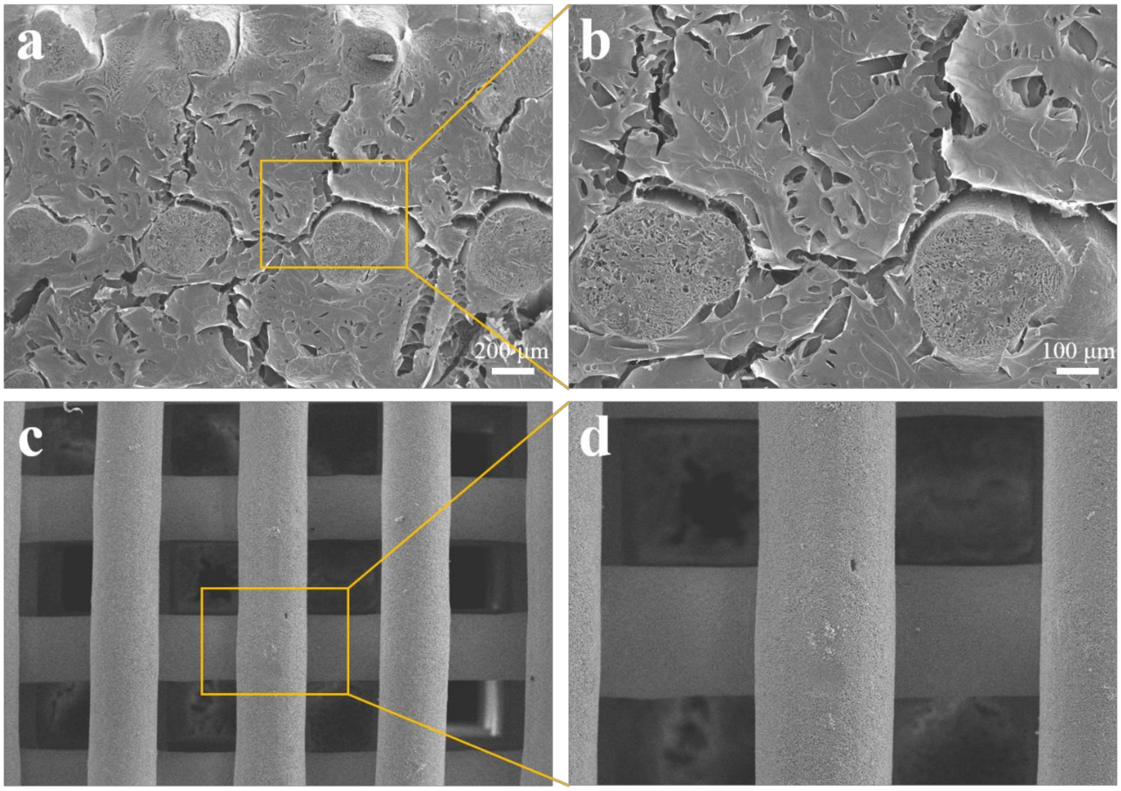

S3. Density values (Hounsfield units) of the scaffolds (Mean ± SD).

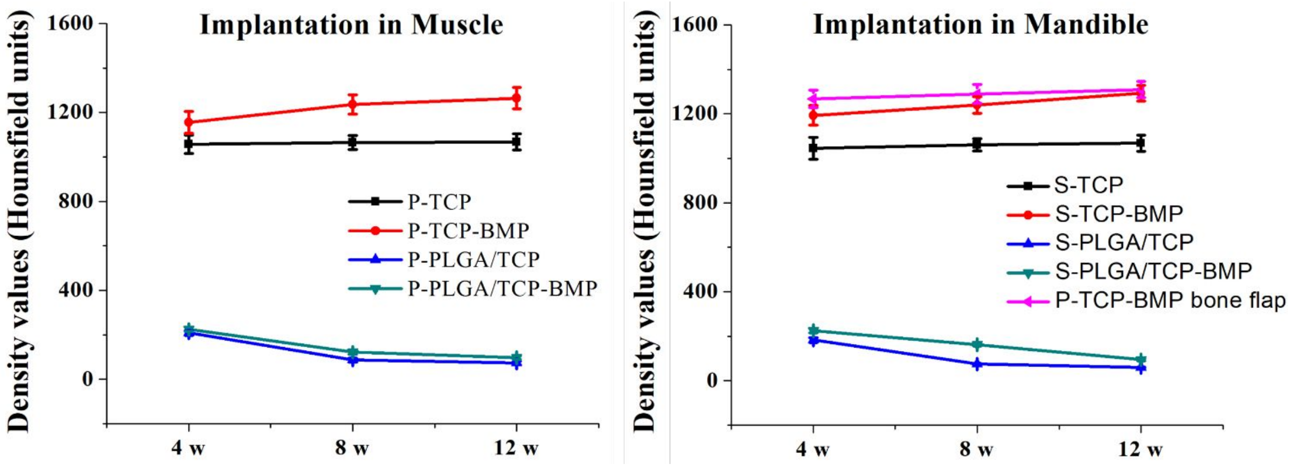

S4. Standardized ^{18}F-FDG uptake values (Mean ± SD) for different scaffolds.

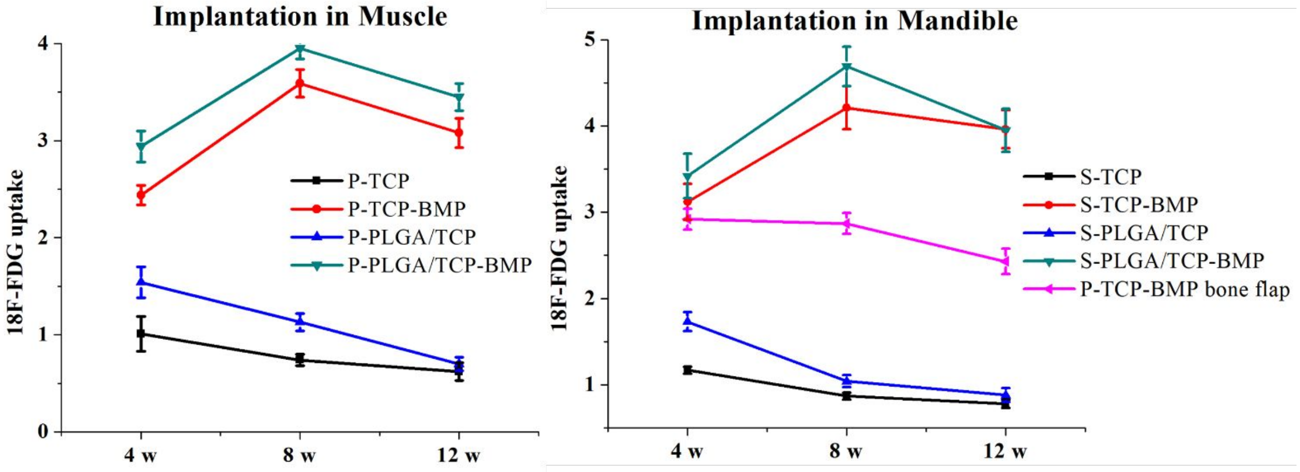

S5. Gross views of ectopically implanted scaffolds after three months. a. PLGA/TCP, b. PLGA/TCP-BMP, c. TCP, d. TCP-BMP.

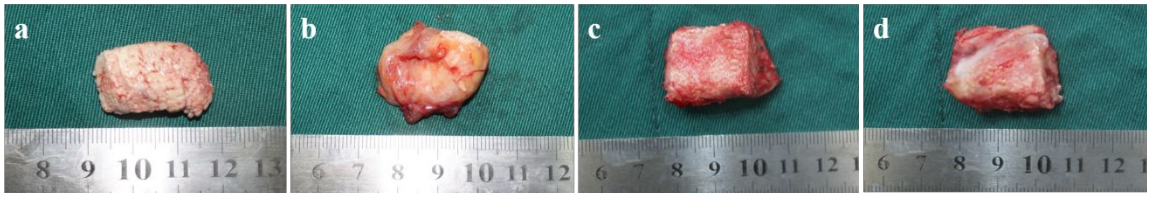

Supplement: Supplementary file 1 — ab1c00509_si_001.pdf [file ab1c00509_si_001.pdf]
